# Supplementary material for: Content-rich biological network constructed by mining PubMed abstracts
Source: BMC Bioinformatics. 2004 Oct 8;5:147. doi: 10.1186/1471-2105-5-147 (PMC528731; doi:10.1186/1471-2105-5-147)
Supplement: Additional File 2 — The original results of the above study (non-essential files are deleted to keep the file size under the limit set by BMC bioinformatics). [file 1471-2105-5-147-S2.bz2 › chilibotAdditionalFile2/dip10/98ID7768895E416/html/SNAP25_STX4A.html]

 


 **SNAP25** and **STX4A** 
  
Found 24 abstracts in PubMed, retrieved 10.  
 

 What does Google say? 
 PDF only 
| .edu only 

---

**Interactive relationship** (e.g. stimulation, inhibition, etc)

**Neutral relationship**- Synaptosome associated 23 kDa protein SNAP23 is a widely expressed isoform of  **SNAP25** , the principal t SNARE of neuronal cells, and colocalizes with syntaxin4  [ **STX4A** ]  in the plasma membrane of 3T3 L1 adipocytes.  Ref: 10713150 J Biol Chem, 2000

**Non-interactive relationship** (e.g. studied together, co-existance, homology, etc.)

- Syntaxin 4  [ **STX4A** ]  and SNAP 25  [ **SNAP25** ]  were also detected.  Ref: 11162495 Biochem Biophys Res Commun, 2001
- A combination of reverse transcription polymerase chain reaction and northern and western blotting is used to show expression of the target membrane SNARE, proteins SNAP 25  [ **SNAP25** ]  and syntaxin 4  [ **STX4A** ]  and the vesicular SNARE protein VAMP, the minimum molecular requirements for core exocytotic complex formation.  Ref: 11472886 Bone, 2001
- However, when vesicle associated membrane protein VAMP 2 was immunoprecipitated from lysates of parotid acinar cells, syntaxin 4  [ **STX4A** ]  and SNAP 23 were not coprecipitated with VAMP 2, although syntaxin 1 and SNAP 25  [ **SNAP25** ] , t SNAREs in neuronal cells, were clearly coprecipitated with VAMP 2 from brain lysates.  Ref: 10739858 Arch Oral Biol, 2000
- Syntaxin 4  [ **STX4A** ]  and SNAP 25  [ **SNAP25** ]  were also detected.  Ref: 11263991 Biochem Biophys Res Commun, 2001
